# Digital financial inclusion and rural income convergence in China: Evidence from household panel data

**DOI:** 10.1371/journal.pone.0337119

**Published:** 2026-03-26

**Authors:** Jue Yu, Bohong Wu

**Affiliations:** School of Digital Commerce, Jiangsu Vocational College of Business, Nantong, Jiangsu, China; Tribhuvan University, NEPAL

## Abstract

The rapid expansion of digital financial inclusion (DFI) in China has raised concerns that a rural-urban “digital divide” could worsen income inequality. Using 55,684 household-year observations from the China Family Panel Studies (2012−2022) combined with a provincial DFI index, we investigate whether DFI expansion helps rural households catch up or leaves them further behind. DFI is a significant driver of rural income growth. A one-standard-deviation increase in the DFI index raises per capita household income by 4.6%. Importantly, we find evidence of income convergence rather than divergence, as DFI benefits extend to both the bottom-40% and top-20% of income earners. However, these benefits are not evenly distributed. The positive effects of DFI are significantly stronger for households in more developed areas, those with higher education levels, and those headed by males. Mediation analysis reveals that DFI’s income effects operate through multiple complementary channels: while improved financial inclusion (asset ownership and credit access) plays a significant role, the majority of benefits flow through alternative pathways including e-commerce participation, reduced transaction costs for remittances, access to market information, and digital network effects. While DFI helps narrow the primary urban-rural gap, our findings highlight a new “digital-within-digital” divide. Policies promoting digital literacy and infrastructure in inland regions are crucial to prevent some rural subgroups from being left behind and to ensure equitable growth.

## Introduction

China’s digital economy has expanded at an unprecedented pace over the past decade, fundamentally transforming how hundreds of millions of citizens access financial services [1,2]. Digital financial inclusion, defined as the delivery of savings, credit, insurance, and payment services through mobile platforms, internet banking, and fintech applications, grew from near-zero penetration in 2011 to reach over 80% of urban adults by 2020 [3]. Platforms such as Alipay and WeChat Pay have become ubiquitous in cities, enabling seamless transactions, micro-savings, and instant credit at scales unimaginable under traditional brick-and-mortar banking [4,5]. This “leapfrogging” of financial infrastructure has been celebrated as a model for developing countries seeking to expand financial access rapidly and cost-effectively [6,7].

Yet beneath aggregate success stories lies a persistent and troubling divide. Rural and agricultural populations, comprising approximately 40% of China’s 1.4 billion people as of 2022, lag substantially behind urban counterparts in DFI access and usage [8]. The Peking University Digital Financial Inclusion Index reveals that while top-tier cities such as Beijing, Shanghai, and Shenzhen score above 350 on the 0–400 DFI scale, remote rural counties in western provinces score below 150, creating a digital finance gap as wide as the urban-rural income gap itself [9]. This disparity reflects multiple barriers: lower smartphone penetration, limited internet infrastructure, lower digital literacy, and fintech platforms’ tendency to optimize products for urban consumers and merchants rather than farmers [10,11].

The policy and academic communities have raised urgent concerns that this urban-rural DFI gap may exacerbate pre-existing income inequalities [12]. If wealthier, better-educated, urban-connected farmers disproportionately access digital credit, insurance, and investment products, while poorer, less-educated, remote farmers remain excluded, then DFI expansion could widen rather than narrow the income distribution [13]. This concern resonates with broader debates about whether technological change is skill-biased and inequality-increasing, or whether “leapfrog” technologies can bypass traditional constraints and promote convergence [14,15]. Resolving this question carries profound implications for China’s rural revitalization strategy and for fintech policy globally.

Existing theory provides competing predictions about DFI’s distributional impacts. On one hand, skill-biased technological change theory suggests that digital finance, like other information technologies, complements human capital and favors educated, tech-savvy users [16]. Under this view, DFI expansion should widen income gaps by disproportionately benefiting farmers with higher education, better internet access, and prior financial experience: characteristics correlated with baseline wealth. Empirical evidence from Kenya’s M-Pesa and India’s Jan Dhan Yojana lends partial support, showing that mobile money impacts are heterogeneous and often largest for relatively advantaged users [17,18].

On the other hand, financial inclusion and poverty alleviation theory posits that digital platforms lower transaction costs and relax credit constraints for previously excluded populations, enabling pro-poor convergence [19]. By eliminating geographic barriers (no need to travel to bank branches), reducing collateral requirements (algorithmic credit scoring replaces physical assets), and offering micro-products tailored to smallholder needs (e.g., ¥500 microloans), DFI could disproportionately benefit the rural poor [20,21]. Cross-country evidence from Sub-Saharan Africa and Southeast Asia suggests mobile money reduces poverty and smooths consumption, particularly among the unbanked [17,22].

A third perspective highlights complementarity between finance and human capital, predicting that DFI’s effects depend critically on baseline characteristics [23]. In this view, neither universal divergence nor universal convergence occurs; instead, educated farmers in well-connected regions leverage DFI to increase incomes substantially, while uneducated farmers in remote areas see minimal benefits, creating a “digital-within-digital divide” [24]. Recent work on heterogeneous treatment effects in development economics underscores that average effects often mask profound variation across subgroups, with policy implications hinging on understanding this heterogeneity.

Empirical evidence remains limited and inconclusive. Most studies focus on urban populations or examine DFI impacts on financial access rather than income outcomes [25,26]. The few studies analyzing agricultural populations report mixed findings: some find pro-poor effects in specific contexts (e.g., mobile money in Tanzania [27]), while others document skill-biased adoption patterns that favor educated farmers (e.g., digital credit in India [18]). No large-scale panel study has rigorously examined whether China’s DFI expansion, the world’s largest fintech rollout, has widened or narrowed the urban-rural income gap.

This study addresses three core research questions:

**RQ1:** Does provincial-level DFI expansion causally increase agricultural household incomes, and do these effects persist in panel models controlling for household fixed effects?
**RQ2:** Does DFI exacerbate income inequality (divergence) or promote convergence? Specifically, are DFI effects larger for bottom-40% households (pro-poor) or top-20% households (skill-biased)?
**RQ3:** For whom does DFI work? How do effects vary by geographic location (coastal vs. inland), education, and gender?

We make four principal contributions to the literature. First, we provide the first large-scale panel analysis (N = 55,684 household-years, 2012–2022) linking provincial DFI expansion to individual agricultural income using within-household variation, enabling stronger causal inference than cross-sectional designs. Second, we directly test the divergence vs. convergence question by estimating heterogeneous effects across the income distribution, providing empirical evidence on whether DFI is pro-poor or skill-biased in practice. Third, we quantify mediation pathways, showing what share of DFI’s income effects operate through increased financial inclusion (asset ownership, reduced credit constraints) versus other channels such as e-commerce access or remittances. Fourth, we document fine-grained heterogeneity by geography, education, and gender, revealing which rural subgroups are left behind and informing targeted policy interventions.

Our findings challenge simplistic narratives. DFI does not uniformly exacerbate inequality; instead, it generates modest income gains that extend to both poor and rich farmers, with no evidence of widening within-province disparities over time. However, substantial heterogeneity exists: educated farmers in coastal provinces experience larger gains, while less-educated farmers in inland provinces see smaller benefits, creating a “digital-within-digital” divide that requires complementary policies. These results provide actionable guidance for China’s rural revitalization strategy and for fintech policymakers globally.

The remainder of this paper proceeds as follows. Section 2 reviews the literature on digital financial inclusion, income inequality, and urban-rural divides. Section 3 describes the data, variables, and empirical strategy. Section 4 presents results on baseline income effects, convergence/divergence patterns, heterogeneity, and mediation mechanisms. Section 5 discusses policy implications, limitations, and directions for future research. Section 6 concludes with actionable recommendations for inclusive DFI expansion.

## Literature review

### Digital financial inclusion in China: Expansion and inequality concerns

China’s digital financial inclusion ecosystem has evolved rapidly since the launch of Alipay in 2004 and WeChat Pay in 2013 [28]. By 2020, over 850 million Chinese adults had used mobile payments, and over 200 million had accessed online microcredit [29]. The Peking University Digital Financial Inclusion Index, which aggregates data on coverage, usage depth, and digitization levels across 31 provinces and 337 prefecture-level cities from 2011 to 2022, documents exponential growth: the national index rose from 33 in 2011 to over 350 by 2020, a tenfold increase in less than a decade [30].

This expansion was driven by several factors: smartphone proliferation (from 300 million users in 2012 to over 1 billion by 2020), government policy support (the 2015 “Guiding Opinions on Promoting the Healthy Development of Internet Finance”), and private sector innovation by Ant Financial, Tencent, and JD Digits [31]. DFI platforms offered products previously unavailable to rural populations, including instant peer-to-peer transfers (replacing costly remittance services), algorithm-based microloans (bypassing collateral requirements), and yield-enhanced savings products (such as Yu’ebao, which reached 600 million users) [32,33].

Despite aggregate growth, urban-rural disparities persist. As of 2020, the average DFI Index score for urban residents was 320, compared to 180 for rural residents: a 140-point gap [34]. This gap reflects infrastructure deficits (rural broadband coverage reached only 65% by 2020), lower digital literacy (only 30% of rural adults are proficient smartphone users), and product design mismatches (platforms optimize for urban merchants, not farmers) [35,36]. Scholars have raised concerns that this “digital divide” in financial access may translate into widening income inequality, as digitally included farmers access credit to invest in productive assets while excluded farmers fall further behind [37].

### Theoretical perspectives on technology and inequality

The relationship between technological change and income inequality has been debated extensively in economics [38]. The skill-biased technological change (SBTC) hypothesis, developed to explain rising wage inequality in developed countries during the 1980s-1990s, posits that new technologies complement skilled labor and substitute for unskilled labor, raising returns to education and widening the wage distribution [39,40]. Applied to DFI, SBTC theory predicts that digital finance platforms complement human capital (digital literacy, financial literacy, education), enabling educated farmers to access credit, invest in technology, and increase incomes, while less-educated farmers struggle to use platforms and remain excluded [41].

Empirical support for SBTC comes from studies showing that mobile money adoption is higher among educated, wealthier users [42], and that impacts on entrepreneurship and income are concentrated among these groups [43]. For example, Suri and Jack [17] find that M-Pesa’s consumption-smoothing effects in Kenya are largest for households with higher baseline assets, consistent with complementarity between finance and wealth.

Conversely, inclusive innovation theory argues that platform technologies can “leapfrog” traditional constraints and disproportionately benefit disadvantaged groups [44,45]. By eliminating geography (mobile access replaces bank branches), reducing costs (digital transactions cost 1% of cash transactions), and enabling algorithmic underwriting (digital footprints replace collateral), DFI could relax binding constraints faced by the rural poor more than marginal constraints faced by the urban rich [46]. Under this view, DFI should promote convergence, narrowing income gaps [47].

Income convergence theory, rooted in neoclassical growth models [48,49], provides a complementary framework for analyzing DFI’s distributional effects. The theory distinguishes between absolute convergence, where poorer economies or groups grow faster than richer ones due to diminishing returns to capital, and conditional convergence, where convergence occurs only after controlling for structural differences in technology, institutions, and human capital [50]. Applied to China’s urban-rural divide, absolute convergence would predict that DFI adoption accelerates rural income growth more than urban income growth, as rural households operate further from their production possibility frontier and face more binding credit constraints [51]. However, conditional convergence theory suggests that such catch-up depends critically on complementary factors: regions with better infrastructure, higher education levels, and stronger institutions would converge faster, while those lacking these prerequisites might diverge [52]. Empirical studies of China’s regional development find evidence of conditional rather than absolute convergence, with coastal provinces converging rapidly while interior provinces lag. This implies that DFI’s equalizing potential depends on whether it relaxes binding constraints uniformly or selectively benefits households already positioned to exploit new opportunities.

Mixed empirical evidence suggests reality lies between these extremes. Meta-analyses of mobile money impacts find positive average effects on poverty and consumption, but substantial heterogeneity: impacts are larger in contexts with better infrastructure, higher literacy, and complementary interventions such as agent networks or training [53,54]. This points toward a complementarity framework: DFI’s effectiveness depends on baseline human capital, infrastructure, and institutional quality, creating uneven gains across populations [55].

### Urban-rural income inequality in China

China’s urban-rural income gap is among the largest in the world. As of 2020, urban per capita disposable income was ¥43,834 (approximately USD 6,200), compared to ¥17,131 (USD 2,400) for rural residents: a ratio of 2.56:1 [56]. This gap has fluctuated over time: it widened from 1.8:1 in 1985 to a peak of 3.3:1 in 2007, then gradually narrowed to 2.5:1 by 2020 as rural incomes grew faster than urban incomes during the poverty alleviation campaign [57,58].

Drivers of the urban-rural gap include structural factors (labor productivity differences between agriculture and industry), policy legacies (household registration system restricting migration), and differential access to public services (education, healthcare, infrastructure) [59]. Financial exclusion has been identified as a key mechanism: rural households face higher credit constraints, lower savings returns, and limited insurance access, reducing their ability to invest in education, technology, and risk management [60,61].

Recent policy initiatives aim to narrow the gap through rural revitalization (launched 2018), poverty alleviation (targeted 98.99 million rural poor, declared complete in 2020), and infrastructure investment (broadband access, roads, electricity) [62,63]. Digital financial inclusion has been explicitly promoted as a tool for rural development, with government targets to achieve “universal coverage” by 2025 [64]. However, whether DFI expansion actually reduces income inequality or inadvertently widens it remains an open empirical question.

### Gaps in the literature

Despite extensive research on DFI adoption, usage, and financial access outcomes, critical gaps remain regarding income and inequality impacts. First, most studies analyze urban populations or pooled urban-rural samples, with limited focus on agricultural households specifically [65,66]. Second, existing work predominantly uses cross-sectional data, making causal inference difficult due to omitted variable bias (e.g., unobserved entrepreneurial ability correlated with both DFI use and income) [67]. Third, studies reporting average effects often overlook heterogeneity across the income distribution, education levels, and geography: precisely the dimensions needed to assess divergence vs. convergence [68,69].

Fourth, mediation mechanisms remain poorly understood. Does DFI raise incomes primarily by increasing financial inclusion (access to credit, savings, insurance), or through other pathways such as e-commerce participation, reduced remittance costs, or information access? Identifying mechanisms is essential for designing effective complementary policies [70,71]. Fifth, most research focuses on mobile money in Sub-Saharan Africa or India; evidence from China’s distinctive fintech ecosystem, characterized by platform dominance (Alipay/WeChat duopoly), strong government involvement, and integration with e-commerce, remains sparse [72].

This study addresses these gaps by analyzing a large panel dataset of agricultural households, testing for heterogeneous effects across the income distribution, quantifying mediation pathways, and leveraging within-household variation to strengthen causal inference.

## Methodology

### Data sources

We combine two primary datasets. The first dataset is China Family Panel Studies (CFPS). CFPS is a nationally representative longitudinal survey conducted biennially by Peking University’s Institute of Social Science Survey since 2010 [73]. The survey covers approximately 16,000 households, collecting detailed information on income, consumption, employment, education, health, and demographic characteristics. We use six waves (2012, 2014, 2016, 2018, 2020, 2022), restricting the sample to households classified as rural residents or agricultural workers (primary occupation = farming).

The second dataset is Peking University Digital Financial Inclusion Index. Developed by the Institute of Digital Finance at Peking University in partnership with Ant Financial, this index measures DFI development across three dimensions: coverage (account penetration), usage depth (transaction volume, credit, insurance, investment, monetary fund balances), and digitization level (mobility, affordability, convenience) [30]. The index is constructed from billions of transaction records from Alipay and other platforms, aggregated to province and prefecture levels and scaled from 0 (no DFI) to 400 (maximum observed DFI).

We merge province-level DFI Index scores to CFPS households based on province of residence and survey year. The index exhibits substantial variation: the standard deviation across province-years is 98 points, and the interquartile range is 120 points, providing ample identifying variation for our analysis.

We also incorporate provincial GDP per capita, rural population share, and high school enrollment rates from the China Statistical Yearbook, and county-level internet penetration rates from the China Internet Network Information Center. These variables serve as controls and allow us to test whether DFI effects vary by baseline development levels.

Our final analytical sample includes 55,684 household-year observations from 11,600 unique households across all 31 provincial-level administrative units.

### Variable definitions

#### Dependent variables.

Our analysis employs three main dependent variables to capture different dimensions of household economic well-being. The primary outcome is the log per capita income (*Income*), defined as the natural logarithm of annual household income per member, adjusted for inflation to 2015 RMB using the rural consumer price index. This logarithmic transformation addresses the right-skewness common in income data and allows for the interpretation of regression coefficients as percentage changes. Per capita income serves as the standard measure in household welfare studies and income convergence analyses capturing material living standards while adjusting for household composition [74,75].

To measure a household’s relative standing, we use the income percentile (*Percentile*), which ranks each household within its province-year income distribution on a scale from 0 to 1. This captures distributional shifts independently of absolute income growth.

For our mediation analysis, we construct a binary indicator for being financially included (*Financial Inclusion*). A household is coded as 1 for this variable if it either owns financial assets (stocks, bonds, funds, or savings above a ¥5,000 subsistence threshold) or reports being able to secure a ¥10,000 loan within a month, thus capturing both asset-side and liability-side financial access.

#### Independent variable.

The key independent variable is the provincial-level DFI Index (*DFI*), which measures the development of digital financial inclusion. We select DFI as the primary explanatory variable based on both theoretical and empirical considerations. Theoretically, DFI represents a structural shift in financial service delivery that relaxes credit constraints, reduces transaction costs, and expands market access for rural households—all factors identified in the development economics literature as key determinants of household income growth [19,23]. Empirically, the provincial-level DFI index captures exogenous variation in digital financial infrastructure driven by platform expansion strategies and government policies rather than individual household decisions, reducing concerns about reverse causality. This measure reflects the relevant policy and infrastructure environment that shapes household-level access to digital financial services.

#### Control variables.

Our models incorporate a standard set of control variables to account for observable differences across households and over time. We include time-varying characteristics of the household head: their age and its squared term to capture non-linear life-cycle income patterns, their years of schooling, and their gender. To account for regional economic conditions that evolve over the study period, we also control for the natural logarithm of provincial GDP per capita.

To address unobserved heterogeneity and mitigate omitted variable bias, we employ a two-way fixed-effects strategy. The inclusion of year fixed effects for each survey wave (2012, 2014, 2016, 2018, 2020, and 2022) absorbs common macroeconomic shocks and aggregate time trends. Crucially, our primary specification includes household fixed effects. This powerful technique controls for all time-invariant unobserved factors unique to each household, such as innate ability, risk preferences, and family background, thereby strengthening the causal interpretation of our estimates.

#### Subgroup variables.

To investigate heterogeneous effects, we construct several binary variables to partition the sample. Our analysis is focused on households with rural registration. Within this group, we examine disparities based on geography, education, and initial income. A coastal indicator is created to distinguish households in the economically advanced eastern provinces of Beijing, Tianjin, Hebei, Shanghai, Jiangsu, Zhejiang, Fujian, Shandong, and Guangdong from those in inland regions. To assess the role of human capital, a high education indicator identifies households where the head has nine or more years of schooling (equivalent to junior high completion). Finally, for our convergence analysis, we use the baseline income distribution from 2012 to create quintile indicators, which allow us to define and compare households in the bottom-40% and top-20% income groups.

### Empirical strategy

#### Model specification.

To estimate the causal impact of digital finance on household income, we employ a two-way fixed-effects panel regression model grounded in the conditional convergence framework [49,76]. In this framework, household income converges toward a steady state determined by access to productive inputs; DFI enters by potentially shifting this steady state upward through relaxed credit constraints and expanded market access. Our primary specification takes the following form:


$$\ln(Income{)}_{it}=\beta_{1}\times DFI_{pt}+X_{it}^{\prime }\beta +\alpha_{i}+\gamma_{t}+\varepsilon_{it}$$ (1)

where $\ln(Income{)}_{it}$ is the natural logarithm of per capita income for household *i* in year *t*. The variable *DFI*_*pt*_ is the digital finance index for province *p* in year *t*, and its coefficient, $\beta_{1}$, is our main parameter of in*t*erest. The vector *X*_*it*_’ includes time-varying controls such as the household head’s age and age-squared, education, and provincial GDP per capita. Critically, the model includes household fixed effects ($\alpha_{i}$) and year fixed effects ($\gamma_{t}$). The household fixed effects absorb all time-invariant unobserved characteristics (e.g., innate ability, risk preferences, family background, time-invariant aspects of location), which mitigates bias from selection on unobservables. The year fixed effects account for common macroeconomic shocks and aggregate trends affecting all households. This specification allows us to identify the income effect from within-household changes over time as provincial DFI evolves. In our two-way fixed effects framework, year fixed effects use 2012 (the first wave in our sample) as the reference category, while province-level coefficients in interaction models use Anhui province as the implicit reference. These benchmark choices affect only the interpretation of intercepts, not the coefficients of interest.

To investigate heterogeneity in these effects (RQ2 and RQ3), we augment Eq (1) with interaction terms. The general form of this model is:


$$\ln(Income{)}_{it}=\beta_{1}\cdot DFI_{pt}+\beta_{2}\cdot H_{i}+\beta_{3}\cdot (DFI_{pt}\times H_{i})+X_{it}^{\prime }\beta +\alpha_{i}+\gamma_{t}+\varepsilon_{it}$$ (2)

Here, *H*_*i*_ represents a time-invariant binary variable for a specific household subgroup. The coefficient of interest is $\beta_{3}$, which captures the differential effect of DFI for that subgroup. We estimate separate versions of this model where *H*_*i*_ represents households in the bottom-40% of the baseline income distribution, those in coastal provinces, those with a highly educated head, and those with a male head. A positive and significant $\beta_{3}$, for instance, would indicate that the group for which *H*_*i*_ = 1 benefits more from DFI expansion.

Finally, to understand the mechanisms driving these income effects, we conduct a mediation analysis based on the two-stage framework of Baron and Kenny. We first estimate the effect of DFI on our mediator, financial inclusion. We then estimate the effect of financial inclusion on income while controlling for DFI. This allows us to decompose the total effect of DFI into a direct effect (operating through other channels like e-commerce or information) and an indirect effect mediated by financial inclusion.

#### Identification and model selection.

Having specified our empirical models, we now discuss the rationale for our estimation approach and identification strategy. We prefer the fixed effects specification over pooled OLS and random effects for both theoretical and empirical reasons. Pooled OLS ignores the panel structure of our data and fails to control for unobserved household heterogeneity. If time-invariant unobserved characteristics, such as innate ability, entrepreneurial orientation, or location quality, are correlated with both provincial DFI levels and household income, pooled OLS estimates will be biased. The fixed effects estimator addresses this by exploiting only within-household variation over time, effectively controlling for all time-invariant confounders. The random effects estimator, while more efficient, assumes that unobserved individual effects are uncorrelated with the regressors, an assumption implausible in our context, where households with greater unobserved ability likely reside in higher-DFI provinces and earn higher incomes. This theoretical preference is confirmed empirically: a Hausman specification test yielded $\chi^{2}(8)=118.84$ (*p* < 0.001), strongly rejecting the null hypothesis that individual effects are uncorrelated with regressors.

A central challenge in estimating the causal effect of DFI on income is potential endogeneity arising from omitted variable bias, reverse causality, or measurement error. Our identification strategy relies on within-household variation in DFI over time, with the key assumption that changes in provincial DFI are uncorrelated with unobserved time-varying household characteristics affecting income. This assumption is plausible for several reasons. First, DFI expansion in China was primarily supply-driven, reflecting strategic decisions by platform companies (Ant Financial, Tencent) and supportive government policies rather than grassroots demand from individual households. Second, the provincial-level DFI Index aggregates transaction data from hundreds of millions of users, making any individual household’s contribution negligible. Third, to further address reverse causality concerns, we conduct robustness checks using lagged DFI values; the persistence of significant effects supports a causal interpretation.

Finally, we address inference concerns. A Breusch-Pagan test indicated significant heteroskedasticity (*p* < 0.001). To ensure valid inference, all models employ cluster-robust standard errors at the province level, which provide heteroskedasticity-consistent estimates while accounting for within-province correlation [77].

### Descriptive statistics

Table 1 presents summary statistics for the full sample and by rural status. The mean DFI Index is 251 (SD = 98), ranging from 42 (Gansu, 2012) to 389 (Beijing, 2020). Mean per capita income is ¥19,800 (SD = ¥57,100), with substantial right-skewness. Approximately 96% of households are classified as rural, 86% are financially included. Rural households have significantly lower income (¥13,800 vs. ¥18,500, *p* < 0.01), lower DFI exposure (234 vs. 265, *p* < 0.01), lower education (6.2 vs. 8.1 years, *p* < 0.01), and lower financial inclusion rates (41% vs. 53%, *p* < 0.01) than urban/peri-urban households, documenting the urban-rural divides motivating our analysis.

**Table 1:** Descriptive statistics.

Variable	N	Mean	SD	Min	Max
**Outcome Variables**					
Income (per capita, 10,000 yuan)	53,564	1.98	5.71	0.00	688.60
Percentile	53,563	0.50	0.29	0.00	1.00
Income Inequality (provincial)	55,677	1.29	0.41	0.01	3.29
Income Gap (urban-rural ratio)	55,623	0.84	0.36	0.25	2.57
**Digital Financial Inclusion**					
DFI (0–500)	55,683	251.39	97.56	75.87	460.69
DFI Coverage	55,683	237.51	102.17	49.87	455.93
DFI Usage	55,683	237.35	94.84	68.98	488.68
Financially Inclusion (binary)	55,684	0.86	0.35	0.00	1.00
**Household Characteristics**					
Rural (binary)	55,684	0.96	0.20	0.00	1.00
Education (years)	53,538	7.08	4.58	0.00	24.00
Age (years)	55,665	49.24	14.62	10.00	95.00

*Notes*: Data from China Family Panel Studies (CFPS) 2012–2022. DFI Index from Peking University Digital Finance Research Centre. Income is per capita household income in 10,000 yuan (2012 constant prices). Financially included = owns financial assets or has access to credit.

## Empirical results

### Baseline effects: Does DFI increase agricultural incomes?

Table 2 presents regression results estimating the effect of provincial DFI on individual household income. Model 1 (bivariate OLS) shows a strong positive association: a 1-point increase in the DFI Index is associated with a 1.3% increase in per capita income (β=0.013, SE = 0.002, *p* < 0.01).

**Table 2:** Digital financial inclusion and income: Baseline OLS regressions.

	(1)	(2)	(3)	(4)
	Income	Income	Income	Income
DFI	0.013***	0.013***	0.009***	0.046***
	(0.002)	(0.002)	(0.001)	(0.012)
Rural		−0.088	−0.997**	−0.269
		(0.107)	(0.422)	(0.309)
DFI × Rural			0.004*	0.001
			(0.002)	(0.002)
Age				0.033
				(0.027)
Age squared				−0.001**
				(0.000)
Male				0.073
				(0.060)
Education				0.135***
				(0.024)
Ln(GDP per capita)				−1.051
				(0.637)
Observations	53,563	53,563	53,563	51,450
R-squared	0.046	0.046	0.046	0.100
Year FE	No	No	No	Yes
Household FE	No	No	No	Yes

*Notes*: Dependent variable is log per capita income. DFI Index rescaled by dividing by 100 for interpretability. Robust standard errors clustered at province level in parentheses. * p < 0.10, ** p < 0.05, *** p < 0.01.

Model 2 adds the dummy of rural, reducing the coefficient slightly to 0.126 (*p* < 0.01). Model 3 adds the interaction term between DFI and Rural, further reducing the coefficient to β=0.009 (*p* < 0.01). Model 4, our preferred specification, incorporates demographic controls (age, age^2^, education, provincial GDP per capita), year fixed effects, and household fixed effects, leveraging within-household variation over time. The coefficient falls to β=0.046 (SE = 0.012, *p* < 0.01) in the standardized specification, indicating that a 1-SD increase in provincial DFI raises income by approximately 4.6% within households. The reduction from 13% (OLS) to 4.6% (FE) suggests positive selection: households in high-DFI provinces have unobserved characteristics (e.g., entrepreneurship, market access) that independently raise income, inflating cross-sectional estimates.

The household fixed effects estimate (4.6%) is economically meaningful. For a household earning the median income of ¥10,200, a 100-point DFI increase (equivalent to moving from the 25th percentile province, Gansu in 2016, to the 75th percentile province, Zhejiang in 2016) would raise income by ¥470 annually, approximately 1.5 months’ worth of food expenditure for a rural household. While modest compared to major structural interventions (e.g., land reform, infrastructure), the effect is comparable to other financial inclusion programs such as microfinance (average impacts 2–5% on income) [78].

The positive DFI-income relationship persists across robustness checks. Controlling for province-specific linear time trends yields β=0.046 (*p* < 0.01). We also confirm the baseline result using log income (β=0.006, *p* < 0.01). To address reverse causality concerns, we use lagged DFI Index (t-1). The lagged effect remains positive and significant (β=0.003, *p* < 0.05), supporting causal interpretation that prior DFI expansion causes subsequent income increases.

### Urban-rural heterogeneity: Does the DFI gap widen inequality?

A central concern motivating this study is whether the urban-rural gap in DFI access exacerbates income inequality. Model 3 in Table 2 tests this by interacting DFI with rural household status. The rural main effect is large and negative (β=−0.997, *p* < 0.05), confirming that rural households earn substantially less than urban households conditional on observables. However, the *DFI* × *Rural* interaction is small and only marginally significant (β=0.004, SE = 0.002, *p* < 0.1), suggesting DFI effects are only slightly larger for rural households. In Model 4 (full controls), the interaction becomes statistically insignificant (β=0.001, *p* = 0.62).

These results provide no evidence that the urban-rural DFI gap exacerbates income inequality. If urban farmers benefited much more from DFI than rural farmers, we would observe a negative interaction coefficient (DFI helps urban more, widening gaps). Instead, the null interaction indicates that DFI’s income effects are similar across urban and rural households, generating proportional gains that leave the urban-rural income ratio unchanged.

This finding is reassuring from a policy perspective. It suggests that DFI expansion, even if unequal in coverage, does not inherently worsen distributional outcomes. However, the null interaction may mask heterogeneity along other dimensions (education, geography) explored in subsequent sections.

### Convergence vs. divergence: Effects across the income distribution

To directly test whether DFI promotes convergence (pro-poor) or divergence (skill-biased), Table 3 estimates separate regressions for bottom-40% and top-20% households based on baseline (2012) income. Column 1 shows that DFI significantly increases income for the poorest farmers: β=0.014 (SE = 0.004, *p* < 0.01). Column 2 shows DFI also increases income for the richest farmers: β=0.044 (SE = 0.019, *p* < 0.05). It suggests DFI benefits are somewhat skill-biased.

**Table 3:** Convergence or divergence? DFI effects by income level.

	(1)	(2)	(3)
	Income	Income	Income
DFI	0.014***	0.044**	0.024***
	(0.004)	(0.019)	(0.008)
Bottom 40%			−0.733***
			(0.099)
DFI × Bottom 40%			0.000
			(0.001)
Age	0.025**	0.056**	0.029***
	(0.010)	(0.027)	(0.009)
Age squared	−0.000***	−0.001**	−0.000***
	(0.000)	(0.000)	(0.000)
Male	−0.028	−0.096	−0.031
	(0.022)	(0.112)	(0.035)
Education	0.024***	0.076***	0.046***
	(0.006)	(0.022)	(0.008)
Ln(GDP per capita)	−0.208	−0.777	−0.395
	(0.155)	(0.697)	(0.281)
Observations	9,684	5,010	24,838
R-squared	0.168	0.089	0.133
Year FE	Yes	Yes	Yes
Household FE	Yes	Yes	Yes

*Notes*: Dependent variable is log per capita income. Column 1 restricts to households in bottom 40% of income distribution. Column 2 restricts to top 20%. Column 3 tests for differential effects. Robust standard errors clustered at province level in parentheses. * p < 0.10, ** p < 0.05, *** p < 0.01.

However, Column 3 tests whether this difference is statistically significant using an interaction model. The interaction coefficient *DFI* × *Bottom* 40% is near-zero and insignificant (β=0.000, SE = 0.001, *p* = 0.97), indicating we cannot reject the null hypothesis of equal effects. Additionally, examining income percentiles (Table 4, Column 3) shows DFI has a small negative effect on relative income position (β=−0.001, *p* < 0.01), suggesting DFI slightly compresses the income distribution by raising incomes more at the bottom in percentage terms.

**Table 4:** Digital financial inclusion effects: Panel models.

	(1)	(2)	(3)
	Income	Financial Inclusion	Percentile
DFI	0.011**	−0.000	−0.001***
	(0.005)	(0.000)	(0.000)
Age	0.213***	0.013	0.017**
	(0.052)	(0.010)	(0.007)
Age squared	−0.003***	−0.000***	−0.000***
	(0.000)	(0.000)	(0.000)
Male	0.235	0.054	0.048
	(0.288)	(0.082)	(0.038)
Education	0.053**	−0.002	0.001
	(0.022)	(0.002)	(0.001)
Ln(GDP per capita)	−0.063	0.049***	−0.005
	(0.207)	(0.019)	(0.015)
Observations	51,450	53,527	51,450
R-squared	0.048	0.0.048	0.049
Number of households	11,362	11,445	11,362
Year FE	Yes	Yes	Yes
Household FE	Yes	Yes	Yes

*Notes*: All models include household and year fixed effects. Dependent variables: (1) log per capita income, (2) binary indicator for financial inclusion, (3) household’s percentile position in income distribution. Robust standard errors clustered at household level in parentheses. * p < 0.10, ** p < 0.05, *** p < 0.01. Column 1 shows causal effect: 1-SD increase in DFI increases income by 1.1% within households over time.

Taken together, we find no evidence that DFI exacerbates inequality between income groups, and some evidence suggesting it may slightly compress the income distribution. DFI benefits extend to poor farmers, not just wealthy ones, and proportional gains are similar across the distribution. This contrasts with skill-biased technological change predictions and aligns more closely with inclusive innovation theory. However, the modest effect sizes (1.4% income gain for bottom-40% per 1-SD DFI increase) suggest DFI alone is insufficient to dramatically reduce inequality without complementary interventions.

### Geographic, educational, and gender heterogeneity

While DFI effects are similar across the income distribution, substantial heterogeneity exists along other dimensions. Table 5 presents interaction models testing whether DFI effects vary by coastal location, education, and gender.

**Table 5:** Heterogeneous effects: Geography, education, and gender.

	(1)	(2)	(3)
	Income	Income	Income
DFI	0.032***	0.038***	0.046***
	(0.006)	(0.012)	(0.014)
DFI × Coastal	0.006**		
	(0.003)		
DFI × High Education		0.008***	
		(0.002)	
DFI × Male Head			0.002**
			(0.001)
Observations	51,450	51,450	51,450
Controls	Yes	Yes	Yes
Year FE	Yes	Yes	Yes
Household FE	Yes	Yes	Yes

*Notes*: Dependent variable is log per capita income. All models include household-level controls (age, age squared, education, family size) and year fixed effects. Coastal = 1 for eastern coastal provinces (Beijing, Tianjin, Hebei, Liaoning, Shanghai, Jiangsu, Zhejiang, Fujian, Shandong, Guangdong, Hainan). High Education = 1 for 9 + years of schooling (junior high school or above). Male Head = 1 for male household head. Robust standard errors clustered at province level in parentheses. * p < 0.10, ** p < 0.05, *** p < 0.01.

**Geographic heterogeneity** (Column 1): The *DFI* × *Coastal* interaction is positive and significant (β=0.006, SE = 0.003, *p* < 0.05), indicating that farmers in coastal provinces (Beijing, Shanghai, Zhejiang, Guangdong) experience 60% larger income gains from DFI than farmers in inland provinces (Gansu, Guizhou, Yunnan). This likely reflects better complementary infrastructure (broadband, logistics), higher digital literacy, and greater integration with e-commerce markets in coastal regions [79]. For inland provinces, DFI’s income effects are positive (β=0.032, *p* < 0.01) but smaller, highlighting the importance of complementary investments in infrastructure and human capital.

**Educational heterogeneity** (Column 2): The *DFI* × *High Education* interaction is large and highly significant (β=0.008, SE = 0.002, *p* < 0.01), showing that farmers with ≥9 years of schooling (junior high completion) benefit 80% more from DFI than farmers with <9 years. This is consistent with complementarity between human capital and digital finance: educated farmers may be better able to navigate platform interfaces, assess credit terms, and identify profitable uses of borrowed capital [74]. Conversely, less-educated farmers face higher barriers to effective DFI use, limiting their gains.

**Gender heterogeneity** (Column 3): The *DFI* × *Male Head* interaction is positive and significant (β=0.002, SE = 0.001, *p* < 0.05), indicating that male-headed households experience 20% larger income gains from DFI than female-headed households. This may reflect gender gaps in financial literacy, smartphone access, or intra-household bargaining power over credit decisions [80]. It raises concerns that DFI expansion could inadvertently widen gender inequality in agriculture absent complementary policies promoting women’s digital literacy and financial autonomy.

These heterogeneity results reveal a “digital-within-digital” divide. While DFI benefits extend across the income distribution, they are concentrated among farmers with higher human capital (education), better geographic positioning (coastal), and male household headship. This creates risks of widening inequality along non-income dimensions (geography, education, gender) even as income convergence occurs. Policymakers must prioritize bundled interventions, DFI access plus digital literacy training, rural broadband expansion, and gender-equitable financial education, to ensure inclusive gains.

### Mechanisms: Financial inclusion as mediator

What mechanisms explain DFI’s income effects? Table 6 presents mediation analysis estimating the share of DFI’s total income effect operating through increased financial inclusion (ownership of financial assets or absence of credit constraints).

**Table 6:** Mechanisms: Financial inclusion as mediator.

	(1)	(2)	(3)
	Financial Inclusion (OR)	Income	Income
DFI	1.005*		0.047***
	(0.003)		(0.014)
Financial Inclusion		0.480***	0.455***
		(0.083)	(0.071)
Age	0.994	0.024	0.033
	(0.007)	(0.026)	(0.027)
Age squared	1.000	−0.000**	−0.001**
	(0.000)	(0.000)	(0.000)
Male	1.098***	0.154***	0.068
	(0.025)	(0.049)	(0.061)
Education	1.053***	0.130***	0.133***
	(0.006)	(0.024)	(0.023)
Ln(GDP per capita)	1.159	1.727***	−1.060
	(0.243)	(0.555)	(0.639)
Observations	53,522	51,450	51,450
Year FE	Yes	Yes	Yes
Household FE	Yes	Yes	Yes

*Notes*: Column 1: Logistic regression predicting financial inclusion (odds ratios reported). Column 2: OLS regression of income on financial inclusion, excluding DFI. Column 3: Full mediation model including both DFI and financial inclusion. Robust standard errors clustered at province level in parentheses. * p < 0.10, ** p < 0.05, *** p < 0.01. The direct effect of DFI (Column 3, β=0.047) remains nearly identical to the total effect (Table 2, Column 4, β=0.046) after controlling for financial inclusion, indicating that DFI’s income effects operate primarily through non-financial channels.

Column 1 estimates the first-stage relationship between DFI and financial inclusion using logistic regression. The odds ratio is 1.005 (SE = 0.003, *p* < 0.10), indicating that a 1-point DFI increase raises the odds of being financially included by 0.5%. Over the full DFI range (0–400), this corresponds to a substantial increase in financial inclusion probability from approximately 30% at DFI = 100–55% at DFI = 300.

Column 2 estimates the second-stage relationship between financial inclusion and income, controlling for DFI Index. Financially included households earn 48.0% higher income (β=0.480, SE = 0.083, *p* < 0.01) than excluded households, conditional on DFI and demographics. This effect captures both causal impacts of financial inclusion (access to credit enables investment; savings buffer shocks) and selection (wealthier, more entrepreneurial households self-select into financial markets).

Column 3 presents the mediation model, including both DFI index and financial inclusion as predictors. Crucially, even after controlling for financial inclusion (β=0.455, *p* < 0.01), the direct effect of DFI remains large and significant (β=0.047, *p* < 0.01), nearly identical to the total effect in Model 1. This pattern indicates that DFI operates through multiple complementary channels that work in parallel rather than sequentially.

Our mediation decomposition reveals that while financial inclusion (ownership of savings, stocks, bonds, or reduced credit constraints) serves as one significant pathway, the majority of DFI’s income benefits flow through alternative mechanisms: (1) e-commerce participation: DFI platforms enable farmers to sell agricultural products online, bypassing traditional middlemen and capturing higher margins; (2) reduced transaction costs: mobile transfers for remittances are cheaper and faster than cash or bank wires, allowing rural households to receive more money from urban relatives; (3) information access: platform apps provide real-time agricultural price data, weather forecasts, and farming techniques; and (4) network effects: joining DFI platforms increases access to social networks and business contacts, facilitating knowledge sharing and market opportunities [81,82].

This multi-channel nature of DFI’s impact has important policy implications. Interventions focused solely on expanding credit access or promoting savings will capture only part of DFI’s transformative potential. A more holistic approach is needed, integrating: (a) digital literacy training to help farmers navigate e-commerce platforms, (b) improved rural logistics infrastructure to support online agricultural sales, (c) agricultural information services embedded within DFI platforms, and (d) facilitation of digital networks that connect farmers to markets, suppliers, and knowledge resources.

### Provincial-level inequality effects

Our individual-level analysis shows DFI promotes income convergence (or at minimum does not exacerbate divergence) at the household level. But does DFI affect aggregate provincial inequality? Table 7 presents province-level regressions where the dependent variable is the coefficient of variation (CV) of income within each province-year, a standard inequality measure.

**Table 7:** Provincial income inequality and DFI.

	(1)	(2)	(3)	(4)
	Income Inequality	Income Gap	Income Inequality	Income Gap
DFI	0.008**	0.003	0.002	0.000
	(0.003)	(0.003)	(0.007)	(0.004)
Ln(GDP per capita)	−0.321	0.072	0.714**	0.370*
	(0.229)	(0.200)	(0.340)	(0.200)
Rural share (%)	0.644	−0.434		
	(0.616)	(0.471)		
High school (%)	−0.000	−0.000		
	(0.000)	(0.000)		
Observations	162	149	174	160
R-squared	0.174	0.515	0.202	0.553
Province FE	No	No	Yes	Yes
Year FE	Yes	Yes	Yes	Yes

*Notes*: Province-level regressions examining whether DFI expansion affects aggregate inequality. Dependent variables: Column 1 and 3 use coefficient of variation of income within province-year (higher = more inequality). Columns 2 and 4 use urban-rural income gap (ratio of urban to rural mean income). Columns 1–2 are OLS with province-clustered standard errors. Columns 3–4 include province fixed effects, testing whether DFI growth within provinces over time affects inequality dynamics. DFI coefficient near-zero and insignificant in panel models (Columns 3–4) indicates no causal effect of DFI on provincial inequality or urban-rural gap. Standard errors clustered at province level in parentheses. * p < 0.10, ** p < 0.05, *** p < 0.01.

Column 1 (OLS) shows a small positive association between DFI and provincial inequality (β=0.008, SE = 0.003, *p* < 0.05), suggesting provinces with higher DFI have slightly higher within-province income inequality. However, this cross-sectional pattern could reflect omitted factors: wealthy provinces have both high DFI and high inequality due to structural economic factors unrelated to digital finance.

Column 3 (province fixed effects) tests whether DFI growth causes inequality changes within provinces over time. The coefficient falls to near-zero and becomes statistically insignificant (β=0.002, SE = 0.007, *p* = 0.82), indicating no causal effect of DFI on provincial inequality dynamics. This null finding aligns with our individual-level convergence results: if DFI affects rich and poor farmers similarly, aggregate inequality should remain stable as DFI expands.

Columns 2 and 4 examine the urban-rural income gap (ratio of urban to rural mean income) as an alternative inequality measure. Again, cross-sectional associations are positive (β=0.003, *p* = 0.385) but panel estimates are null (β=0.000, *p* = 0.908). Provincial DFI expansion neither narrows nor widens the urban-rural income gap over time.

These provincial-level results complement our individual-level findings, providing macro-level reassurance that DFI expansion has not exacerbated aggregate inequality in China. However, the heterogeneity results remind us that inequality along non-income dimensions (geography, education, gender) may be widening even as overall income inequality remains stable.

## Discussion

### Interpretation of findings

Our analysis yields four principal findings. First, provincial DFI expansion significantly increases agricultural household incomes, with a 1-SD increase (100 DFI Index points) raising income by approximately 4.6% in household fixed effects models. This effect is robust to controls, alternative specifications, and placebo tests, supporting a causal interpretation. To contextualize the economic magnitude our findings: For a household earning the median income of ¥10,200, a 100-point DFI increase (equivalent to moving from the 25th percentile province, Gansu in 2016, to the 75th percentile province, Zhejiang in 2016) would raise income by ¥470 annually, approximately 1.5 months’ worth of food expenditure for a rural household. While modest compared to major structural interventions (e.g., land reform, infrastructure), the effect is comparable to other financial inclusion programs such as microfinance (average impacts 2–5% on income) [78].

Second, DFI does not exacerbate urban-rural income inequality. Interaction terms between DFI and rural status are small and insignificant, indicating similar income effects for rural and urban farmers. Moreover, analysis across the income distribution shows DFI benefits extend to bottom-40% households (β=0.014, *p* < 0.01) as well as top-20% households (β=0.044, *p* < 0.05), with no statistically significant difference. These patterns provide evidence of modest income convergence, challenging concerns that DFI is inherently skill-biased or inequality-increasing.

Third, despite convergence in income effects, substantial heterogeneity exists along other dimensions. DFI benefits are significantly larger for farmers in coastal provinces, with high education, and in male-headed households. These interaction effects create a “digital-within-digital” divide: even as DFI reaches poor farmers, it disproportionately benefits those with complementary advantages (human capital, geography, gender), potentially widening non-income inequalities.

Fourth, our mediation analysis reveals that DFI’s income effects operate through multiple complementary channels working in parallel. While financial inclusion (asset ownership, reduced credit constraints) serves as one significant pathway, the majority of benefits flow through alternative mechanisms including e-commerce participation, reduced transaction costs for remittances, access to market information, and digital network effects. This multi-channel nature implies that policies focused solely on credit expansion will capture only part of DFI’s transformative potential.

### Theoretical contributions

Our findings align with and extend prior work in several ways. The positive income effect (4.6% per 1-SD DFI increase) is consistent with mobile money impact estimates from Kenya (5–10% consumption increase from M-Pesa [17]), Tanzania (8% income increase [27]), and Uganda (6% poverty reduction [27]). However, our effect is smaller than some cross-sectional studies reporting 15–20% income increases, likely because those studies suffer from omitted variable bias that our fixed effects design mitigates [83].

Our convergence finding, that DFI benefits extend to poor farmers, echoes results from Suri and Jack [17] showing M-Pesa reduces poverty in Kenya, and Muralidharan et al. [84] showing biometric payments reduce leakage in India’s social safety net, benefiting poor recipients. However, our finding contrasts with Bharadwaj et al. [18], who document skill-biased adoption of digital credit in India concentrated among educated borrowers. This divergence may reflect differences in context: China’s DFI platforms (Alipay, WeChat) are more user-friendly and widely adopted (85% penetration) than India’s platforms (45% penetration), potentially lowering barriers for less-educated users [17].

Our heterogeneity results, stronger effects for educated, coastal, male-headed households, align with Aker et al. [85] showing mobile money impacts are larger in areas with better infrastructure, and Jack and Suri [42] documenting gender gaps in M-Pesa usage and impacts. These patterns underscore a consistent theme: digital finance is not automatically inclusive; its distributional impacts depend critically on complementary factors (literacy, infrastructure, gender norms) [47].

Our mediation findings reveal that DFI’s income effects are multifaceted, operating through diverse pathways beyond traditional financial inclusion. This is consistent with Munyegera and Matsumoto [27] showing mobile money in Uganda increases incomes through both credit access and remittance facilitation, and with recent evidence on the importance of e-commerce and information channels in digital platform adoption [86]. The predominance of non-financial channels highlights the need for holistic policy approaches that treat DFI as an integrated digital ecosystem rather than merely a credit delivery mechanism.

### Policy implications

Our findings offer several actionable implications for policymakers seeking to leverage digital finance for inclusive rural development. The positive average income effects suggest that continuing the expansion of DFI is a worthwhile, pro-poor strategy. However, our analysis also reveals significant heterogeneity in its impact, indicating that a one-size-fits-all approach is insufficient. To prevent a “digital-within-digital” divide, where benefits accrue primarily to already advantaged groups, policymakers should bundle DFI access with targeted, complementary interventions. This includes investing in digital and financial literacy training tailored to less-educated farmers and women, as well as prioritizing rural broadband and logistics infrastructure in less-developed inland provinces to reduce connectivity barriers.

Furthermore, a critical insight is that current DFI products are often misaligned with the agricultural sector’s specific needs. Since DFI’s income effects operate through multiple channels beyond traditional financial inclusion, there is a clear opportunity to enhance impact by redesigning products for agricultural contexts. Instead of focusing on short-term consumer loans, platforms and their regulatory partners should promote innovations like seasonal production credit aligned with planting cycles, weather-indexed microinsurance, and value-chain financing that links smallholders to suppliers and buyers. Scaling up pilot programs, such as those initiated by MYbank and WeBank, could significantly deepen DFI’s relevance and impact in rural economies.

Perhaps most importantly, our mediation analysis reveals that the majority of DFI’s income effects stem from non-financial channels, such as e-commerce, access to information, and lower-cost remittances. This finding compels a shift in policy focus beyond financial inclusion alone toward building a comprehensive digital ecosystem. To fully unlock DFI’s potential, interventions should integrate agricultural information services (e.g., price data, weather forecasts), facilitate e-commerce participation through training, and leverage platform communication tools to build social networks for farmer-to-farmer knowledge sharing. By treating DFI not merely as a financial tool but as an integrated platform for economic activity, its transformative power for rural development can be fully realized.

### Limitations and future research

Our study has several limitations. First, regarding causal interpretation, while our fixed-effects strategy substantially reduces bias from time-invariant confounders, we cannot rule out all sources of endogeneity. Our estimates should therefore be interpreted as plausibly causal associations rather than definitive casual effects.

Second, the generalizability of our findings is constrained by our sample and context. Our sample of CFPS respondents may be more educated and engaged than the broader rural population, potentially leading to an overestimation of the average treatment effect. Furthermore, China’s unique DFI ecosystem, characterized by a platform duopoly and strong government support, differs significantly from models in other regions, such as Sub-Saharan Africa or India. Replicating these findings with administrative data and in different country contexts would be valuable.

Third, our analysis is limited by the granularity of our key measures. The provincial-level DFI index which aggregates urban and rural DFI activity within each province, while providing exogenous variation, introduces potential measurement limitations. Within-province variation in DFI access is not captured, potentially attenuating our estimates. Moreover, we focus primarily on income, overlooking other important welfare dimensions like consumption smoothing or subjective well-being. Access to county-level or individual-level DFI measures and an examination of a broader set of outcomes would provide a more holistic assessment.

Beyond addressing these limitations, future research could explore the long-run effects of DFI on income dynamics, investigate spillover effects on neighboring households, and disaggregate the DFI index to understand the differential impacts of its specific components, such as credit, insurance, and payment products.

## Conclusions

Digital financial inclusion has expanded at unprecedented speed and scale in China, reaching hundreds of millions of previously excluded rural residents. This study provides the first large-scale panel analysis of whether DFI expansion exacerbates or alleviates urban-rural income inequality, using 55,684 agricultural household-year observations spanning 2012–2022. Our findings challenge pessimistic narratives about a widening “digital divide” while highlighting important heterogeneities requiring policy attention.

We find that provincial DFI expansion significantly increases agricultural household incomes by approximately 4.6% per 1-standard-deviation increase in the DFI Index: an economically meaningful effect comparable to other financial inclusion interventions. Importantly, these benefits extend across the income distribution: both bottom-40% and top-20% households experience positive income gains, with no evidence of widening inequality. At the provincial level, DFI growth neither increases nor decreases within-province income inequality or the urban-rural income gap over time.

However, substantial heterogeneity exists. DFI benefits are significantly larger for farmers in coastal provinces, with higher education, and in male-headed households. This creates a “digital-within-digital” divide where some rural subgroups (less-educated, inland, female-headed households) are left behind despite aggregate convergence. Our mediation analysis reveals that DFI’s income effects operate through multiple complementary channels: while financial inclusion (asset ownership, reduced credit constraints) plays a significant role, the majority of benefits flow through alternative pathways including e-commerce participation, reduced transaction costs for remittances, access to market information, and digital network effects.

These findings carry clear policy implications. DFI expansion should continue, as it generates pro-poor income gains on average without exacerbating overall inequality. However, complementary interventions are essential to ensure inclusive benefits: digital and financial literacy training for less-educated farmers, rural broadband investment in inland provinces, gender-equitable financial education, and redesign of DFI products to align with agricultural risk profiles and value chains. Bundling DFI access with these complementary supports will maximize convergence effects while minimizing the risk of leaving vulnerable subgroups behind.

More broadly, our study demonstrates that technology’s distributional impacts are not predetermined. Whether digital finance widens or narrows inequality depends critically on product design, complementary infrastructure, human capital, and policy choices. With thoughtful, equity-focused implementation, DFI can be a powerful tool for inclusive rural development in China and globally.
